# Incomplete ileus and hemafecia as the presenting features of multi-organ involved primary systemic AL amyloidosis: a rare case report

**DOI:** 10.1186/s12876-017-0628-3

**Published:** 2017-06-05

**Authors:** Li Tian, Anliu Tang, Xian Zhang, Zhen Mei, Fen Liu, Jingbo Li, Xiayu Li, Feiyan Ai, Xiaoyan Wang, Shourong Shen

**Affiliations:** The Third Xiangya Hospital of Central South University, 138 Tongzipo Road, Yuelu District Changsha, Hunan, 410013 China

**Keywords:** AL amyloidosis, Congo red stain, Bone marrow biopsy, Immunofixation

## Abstract

**Background:**

AL Amyloidosis is known to be a systemic disease affecting multiple organs and tissue while it’s rare that patients present with gastrointestinal symptoms at first and later develop multiple-organ dysfuction. Clinical signs are not specific and the diagnosis is rarely given before performing immunofixation and endoscopy with multiple biopsies. We would like to emphasize the value of precise diagnostic process of AL amyloidosis.

**Case presentation:**

In this case report, we describe a 56-year-old man who presented with recurrent periumbilical pain for 4 months and gradually worsened over a month. After a series of tests, he was finally diagnosed with primary systemic AL amyloidosis. He was treated with a chemotherapy regimen (Melphalan, dexamethasone and thalidomide) achieving a good clinical response.

**Conclusion:**

On account of the high misdiagnosis rate, establishing the most precise diagnosis in first time with typing amyloidogenic protein becomes increasingly vital. Although the presenting feature is usually nonspecific, AL amyloidosis ought to be considered when multiple organs are involved in a short period.

## Background

AL amyloidosis is usually a systemic disease characterized by multiple organs and tissue involvement. Actually the incidence of amyloidosis is not well reported, but probably falls between 5 and 13 per million per year. Prevalence data are also scare, a study in UK reported about 20 per million local people [1]. AL amyloidosis with gastrointestinal tract involvement is usually found on postmortem examination, but less than 1% of these patients have disease symptoms [2]. Moreover, it is uncommon that one patient develops AL amyloidosis with more than three systems involved. Notably, once the heart is affected, the prognosis will be poor [3]. Here, we report a rare case of primary systemic AL amyloidosis that firstly presented with incomplete ileus and hemafecia and later developed systemic symptoms involving more than three organs. We highlight the importance of considering amyloidosis as a possible cause if the patient has prior unexplained gastrointestinal symptoms.

## Case presentation

A 56-year-old man was admitted to our hospital with recurrent periumbilical pain, hemafecia and vomiting for 4 months and gradually worsened over a month. The patient accepted an appendicectomy about 20 years ago, and he had smoked one pack of cigarette per day for about 40 years. He denied any hereditary disease in his family and had no psychosocial history and other medical treatments history.

**Fig. 1:**
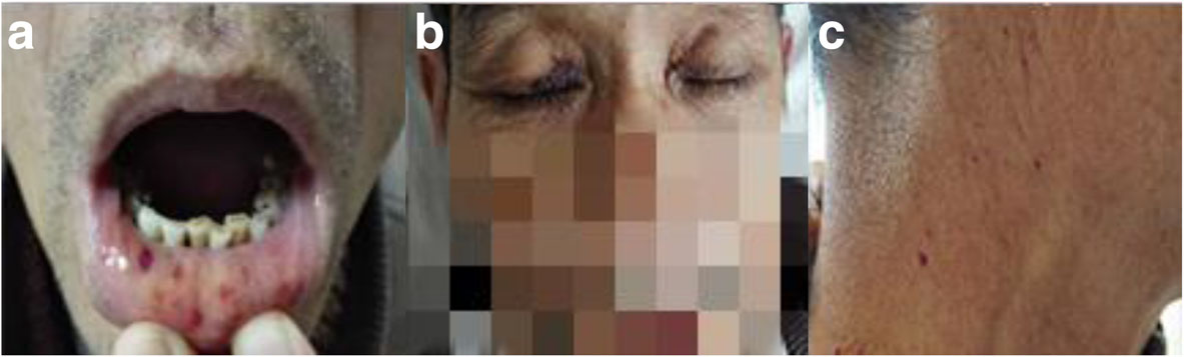
Multiple petechiae and ecchymoses lesions around the eyelids (**b**), on the oral mucosa (**a**) and the neck bilaterally (**c**)

On physical examination, his body temperature, blood pressure, respiratory rate and pulse were 36.6 °C, 124/86 mmHg, 20 bpm, 80 bpm respectively. Multiple petechiae and ecchymoses lesions could been found around the eyelids, on the oral mucosa and the neck bilaterally (Fig. 1). Other pertinent findings included hypoactive bowel sounds and abdominal tenderness and rebound tenderness especially in the right lower quadrant.

Laboratory tests showed a mild anemia and an increased value of erythrocyte sedimentation rate with an elevated C-reactive protein. A normal albumin level and an increased creatinine were revealed in liver and kidney function tests. Coagulation parameters, serum electrolytes, glucose and lipid profile and hyperthyroidism series were all within normal limits. Other tests (Table 1) such as microbiologic and parasitologic analyses of the stools, T-SPOT.TB, bone biopsy and Bence-Jones protein test were all negative. N-terminal prohormone of brain natriuretic peptide (NT-proBNP) reached the level of 2269.4 pg/ml while troponin and myocardial enzymes were normal.

**Table 1:** Laboratory data on first visit to the Third Xiangya Hospital of Central South University

Component	Result	Normal values
Hemoglobin, g/L	114	120-150
Erythrocyte sedimentation rate, mm/h	20	0-15
C-reactive protein, mg/l	9.5	0.068-8.2
Albumin, g/l	36.7	32-48
Creatinine, umol/l	106	44-88
Stool examination for Salmonellae and Shigellae	(−)	(−)
T-SPOT.TB	(−)	(−)
Connective tissue series	(−)	(−)
MPO + PR3 antibodies test	(−)	(−)
Complement 3/complement 4	(−)	(−)
Bone biopsy	(−)	(−)
Bence-Jones protein	(−)	(−)
NT-proBNP, pg/ml	2269.4	<400

*Abbreviation:* (−) negative

**Fig. 2:**
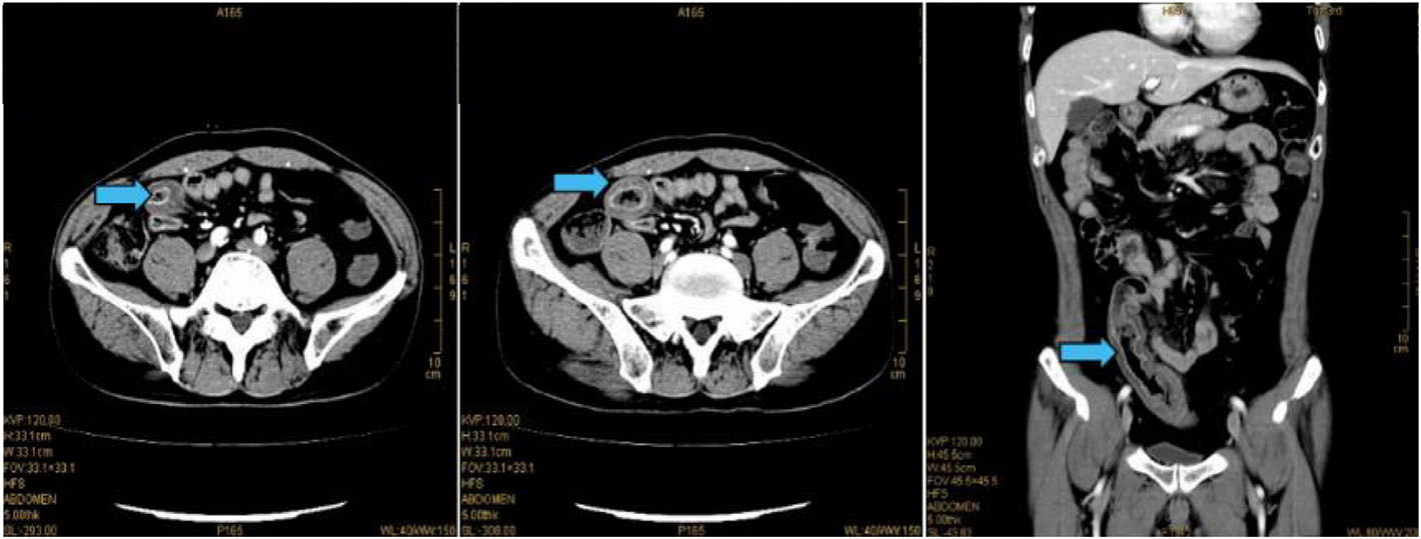
CT scan of the abdomen revealed pelvic ascites with segmental intestinal wall thickening at terminal ileum

**Fig. 3:**
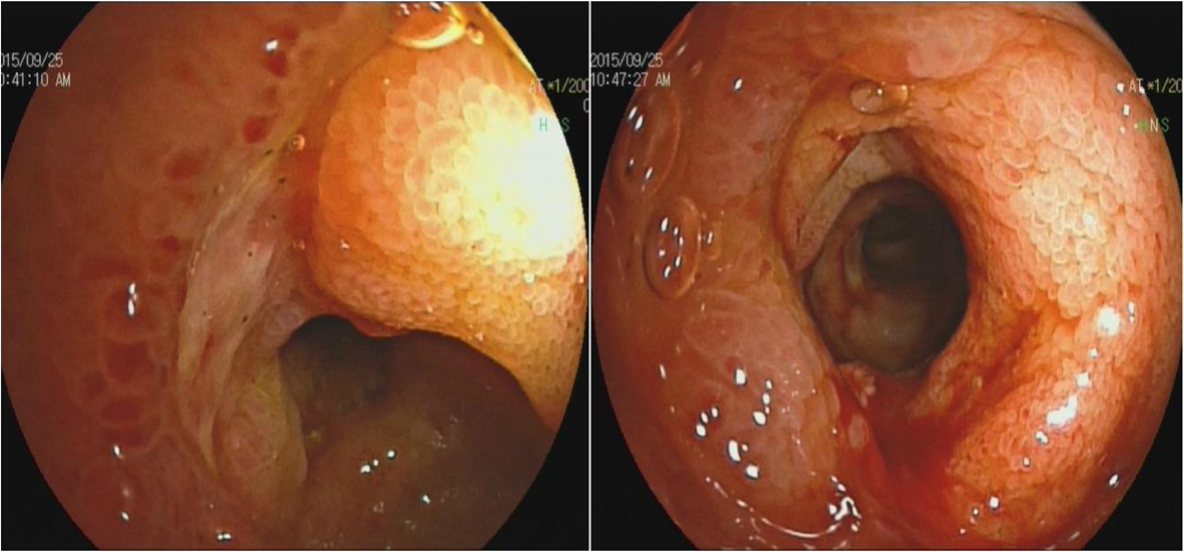
Enteroscope found stricture at terminal ileum caused by semi-annular ulcers

A contrast-enhanced computed tomography (CT) scan of the abdomen revealed pelvic ascites with segmental intestinal wall thickening at terminal ileum, which was about 12 cm in length (Fig. 2). Enteroscope found stricture at terminal ileum caused by semi-annular ulcers (Fig. 3).

**Fig. 4:**
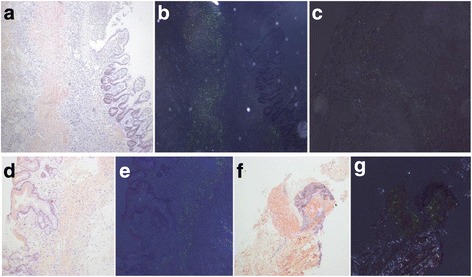
Histopathology of resected ileum showed orange-red positivity with Congo Red staining (**a**); Histopathology of resected ileum with Congo Red staining showed characteristic apple-green birefringence under polarized light (**b**); Histopathology of resected ileum with Congo Red staining showed characteristic apple-green birefringence under polarized light after potassium permanganate pretreatment (**c**); Microscopic findings of the stomach biopsy and orange-red positivity with Congo Red staining (**d**); Characteristic apple-green birefringence under polarized light (**e**); Microscopic findings of the skin biopsy and orange-red positivity with Congo Red staining (**f**); Characteristic *apple-green* birefringence under polarized light (**g**)

Considering all these available data, the patient received symptomatic and supportive treatment. However, his symptoms were aggressive and he had active gastrointestinal bleeding necessitating an exploratory laparotomy and resection of pathological ileum. Histological examination of the resected specimen revealed amorphous, pink deposits in mucosa and vascular walls with a positive Congo red staining (Fig. 4a) and characteristic apple-green birefringence under polarized light (Fig. 4b). Moreover, the deposits were positive for Congo red staining after potassium permanganate pretreatment (Fig. 4c). According to all available information and the analysis of this patient, we made a tentative diagnosis of AL amyloidosis.

Further investigations were performed to make it more explicit. Urine protein electrophoresis revealed increased concentration of β2-microglobulin (4.68μg/ml, norm 0.039-0.169μg/ml) and λ light chains (84.89 mg/l, norm5.71-26.3 mg/l) and a decreased concentration of κ light chains (0.01 mg/l, norm 3.3-19.4 mg/l). Nephrotic range proteinuria of 216 mg (norm: 0-150 mg/day) occurred in 24-h urine collection. Immunofixation demonstrated it as lambda type monoclonal protein in serum specimens. Bone marrow biopsy didn’t reveal any abnormalities.

Biopsy specimens from both the stomach and skin proved the presence of amyloid deposits in the Congo Red staining or under polarized light (Fig. 4d–g). Further immunohistochemistry of gastric specimens detected both λ and κ light chains.

**Fig. 5:**
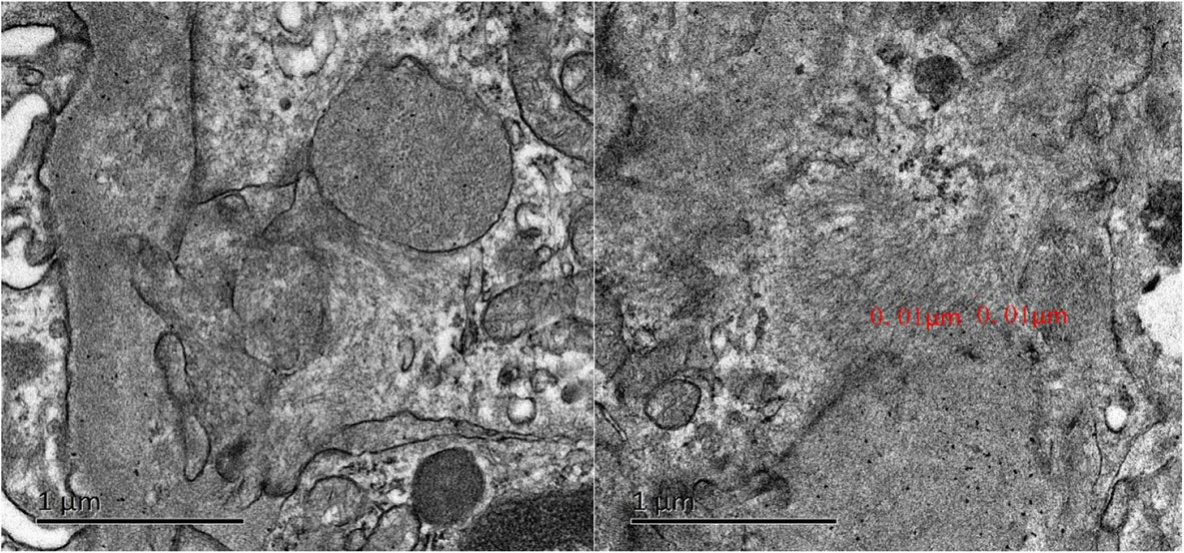
Amyloid materials which are ~10 nm in diameter was showed in renal specimen by electron microscopy

**Fig. 6:**
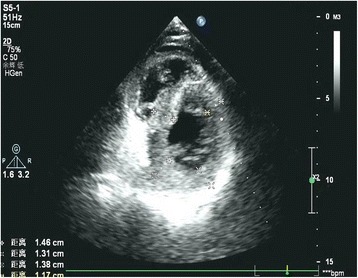
The echocardiographic study disclosed left ventricular thickening, “snowstorm” appearance of the myocardium and a small amount of excessive pericardial fluid

Biopsy from the kidney revealed some brick red deposits in walls of several small blood vessels with Congo red staining. In addition, a small amount of amyloid materials which are ~10 nm in diameter was showed by electron microscopy (Fig. 5). The echocardiographic study disclosed left ventricular thickening and a small amount of excessive pericardial fluid (Fig. 6).

The final diagnosis was primary systemic AL amyloidosis. He was treated with a chemotherapy regimen (Melphalan, dexamethasone and thalidomide) and received a good clinical response.

## Discussion

Amyloidosis is classified as either systemic or limited forms. In localized amyloidosis, deposits are limited to a single organ, while systemic amyloidosis usually affects several organs. But it is less apt to present with gastrointestinal symptoms and involve more than three organs in systemic amyloidosis like our patient.

Kidney is the most frequently affected organ, where the amyloid deposits in the renal glomerulus and interstitial tissue resulting in proteinuria, nephritic syndrome and renal failure [4]. Cardiac amyloidosis manifests itself by a circulatory failure, and the typical light chain amyloidosis of the heart can be revealed by the ECG and ECHO [5]. As for gastrointestinal amyloidosis, small bowel is the most commonly affected site, followed by stomach, colon and esophagus [6]. Endoscopic features of AL amyloidosis involving gastrointestinal tract are not specific including thickened folds, erosions, ulcerations and edema [7–9]. With skin involvement, flexural areas such as eyelids, retroauricular region and the neck, are sites of predilection [10].

Bone marrow plasma cells (BMPC) are responsible for fibril production in primary systemic amyloidosis; nevertheless, the clonality of BMPC is usually not apparent [11]. But detecting monoclonal light chain in serum or urine using immunofixation is more sensitive than biopsy of bone marrow and others [12]. For these patients with normal bone marrow biopsy, just like our case, serum or urine immunofixation and biopsy of the affected organs were appropriate next steps.

In tissue biopsy, samples usually undergo Congo red staining, which display characteristic dichroism and apple green birefringence under polarized light [13]. Congo red dye is the most usual and effective way applied to a tissue which was suspected of amyloid protein accumulation. Usually, we try to get a tissue sample directly from the diseased organ for high reliability of biopsy procedure as our expected. While we must pay attention to avoid the fact that the biopsy of parenchymal organs poses in particular a risk of internal bleeding.

If a patient is referred to amyloisosis, it is important to ensure that the amyloidosis is primary or secondary. Patients are considered with secondary AA amyloidosis caused by a well-defined rheumatic disorder, tuberculosis, multiple myeloma, long-standing inflammatory, symmetrical polyarthritis and connective tissue disorders [14]. Therefore some lab examinations are necessary for differential diagnosis.

Last century, Wright et al. [15] described a simple direct method to further differentiate between primary AL and secondary AA. It shows that secondary AA looses its affinity for Congo red after incubation with potassium permanganate. We applied the method according to Wright on histologic material from our files in order to make the diagnosis of primary AL amyloidosis.

Therapies for primary systemic amyloidosis involves stem cell transplantation and chemotherapy. Even though the former technique provided higher responses and survival prolongation, only a fraction of patients could meet its criteria. Melphalan and prednisone, as well as single-agent dexamethasone were effective treatment for the majority of patients ineligible for stem cell transplantation [16, 17]. As several novel agents appeared, the combination of thalidomide, Melphalan and dexamethasone still tends to result in optimal clinical response [18].

The prognosis of the disease was primarily determined by cardiac involvement. Median survival time of patients with cardiac amyloidosis was significantly shorter than those without cardiac amyloidosis (21 vs. 76 months, *P* < 0.001) [19]. Serum N-terminal portion of natriuretic peptide type B (NT-proBNP) is a highly sensitive marker of myocardial dysfunction and a powerful prognostic determinant in respect to survival for AL patients [20, 21].

## Conclusion

In summary, clinical manifestations of primary systemic amyloidosis are diverse and nonspecific. It should be considered in patients with gastrointestinal symptoms, especially accompanying with multisystemic involvement. Considering the poor survival rate of untreated patients with amyloidosis, immunofixation and biopsy with Congo Red staining are of great diagnostic significance.

## Abbreviations

BMPC: Bone marrow plasma cells
CT: Computed tomography
ECG: Electrocardiography
ECHO: Echocardiographic
NT-proBNP: N-terminal prohormone of brain natriuretic peptide
